# Can Electrochemical Sensors Be Used for Identification and Phylogenetic Studies in Lamiaceae?

**DOI:** 10.3390/s21248216

**Published:** 2021-12-08

**Authors:** Da Wang, Dongling Li, Li Fu, Yuhong Zheng, Yonghua Gu, Fei Chen, Shichao Zhao

**Affiliations:** 1 Key Laboratory of Novel Materials for Sensor of Zhejiang Province, College of Materials and Environmental Engineering, Hangzhou Dianzi University, Hangzhou 310018, China; wangda@hdu.edu.cn (D.W.); feichen@hdu.edu.cn (F.C.); zhaoshichao@hdu.edu.cn (S.Z.); 2 The Jiangsu Provincial Platform for Conservation and Utilization of Agricultural Germplasm, Institute of Botany, Jiangsu Province and Chinese Academy of Sciences (Nanjing Botanical Garden Mem. Sun Yat-Sen), Nanjing 210014, China; lidongling@cnbg.net (D.L.); zhengyuhong@cnbg.net (Y.Z.); g_yhua@aliyun.com (Y.G.)

**Keywords:** electrochemical sensor, Labiatae, plant identification, fingerprints, plant phylogeny

## Abstract

Electrochemical sensors have shown potential in recent years for plant species identification and phylogenetic studies. These works have been used to investigate the affinities of different species in many genera. However, the ability of electrochemical sensors to study relationships between different genera within a family has not been investigated. In this work, we selected 31 species in the Labiatae and 5 exotaxa as subjects to investigate the feasibility of electrochemical sensors at the genus level. The results show that electrochemical sensors are still very effective for the identification of these plants. Different pattern recognition techniques can make the identification more efficient. Also, the fingerprint profiles collected by the sensors can be used for phylogenetic studies of Labiatae. The phylogram divides all the species into five clusters, where the exotaxa are in one cluster. Species in the Labiatae are mainly distributed in four other clusters. Importantly, the different genera of species all showed close affinities, representing that electrochemical fingerprinting can well distinguish the affinities between the different genera. The results of this work demonstrate the great potential of electrochemical sensors in the study of plant phylogeny. Its application is not limited to the study at the species level, but can be extended to the genus level.

## 1. Introduction

Electrochemical sensors capture the electrochemical oxidation and reduction signals of electrochemically active substances in a sample. This sensing technique can be used for the highly sensitive detection of a target substance and also for the recording of the overall electrochemically active profile in the sample. The use of electrochemical techniques for the recording of electrochemically active substances in plant tissues has been shown to be useful for species identification and species level phylogenetic studies [1,2,3]. Electrochemical fingerprinting of plant tissues fixed on the electrode surface can be performed using voltammetry of immobilized particles [4,5,6]. Alternatively, electrochemical fingerprinting of extracts from plant organs can be performed using suitable extraction techniques. So far, electrochemical sensors have been successfully used for species identification and phylogenetic studies, such as *Lycoris* [7], *Pueraria* [8], *Chimonanthus* [9], and *Taxodium* [10]. These methodological explorations have focused on the study of relationships between different species within a genus. In order to validate the feasibility of electrochemical fingerprinting, the selection of these genera tends not to include particularly complex phylogenetic situations. Among these carefully selected research targets, the fingerprint profiles collected by electrochemical sensors demonstrate extraordinary results. Fingerprint profiles can be used, not only for the identification of different species, but also for the study of their affinities. The signals recorded by electrochemical fingerprinting provide the types and relative amounts of electro-active substances in plant tissues. Because the type and amount of compounds in plants are controlled by genes, plants with close relationship have similar composition distribution, while plants with distant relationship have great differences. The analysis of electrochemical signal differences can reflect the phylogenetic position of plants. The results of previous studies have shown that electrochemical fingerprinting-based phylogenetic investigations supported some molecular phylogenetic studies. Although we believe that the use of fingerprint profiles collected by electrochemical sensors for other genus studies still needs to be carried out, the time has come to verify the feasibility of this methodology for the investigation of affinities between different genera. Therefore, we attempted in this work to use fingerprint profiles collected by electrochemical sensors for the identification and phylogenetic study of Labiatae.

Lamiaceae is one of the more widely distributed families worldwide, with about 220 genera and more than 3500 species [11]. Labiatae are mostly one to perennial herbs, with occasional semi-shrubs, shrubs, trees or vines. Their roots are fibrous or fusiform, very occasionally small tubers. Their stems are often quadrangular, with occasional aerial walking stems or underground stolons. Labiatae are of great economic value due to the presence of volatile oils [12]. It includes those used as flavorings such as *Mentha haplocalyx*, *Thymus mandschuricus*, *Lavandula pedunculata*, and *Ocimum basilicum*. The Labiatae also contain important aromatic oil plants, such as *Leonurus sibiricus*, *Agastache rugosa*, *Salvia miltiorrhiza*, *Perilla frutescens* and *Elsholtzia ciliate*. The most widely influential taxonomic system of the Labiatae is that of J. Briquet [13]. This system has been widely accepted by the taxonomic community since its publication in 1892. His system is divided into two major groups based on whether or not the style is attached to the base of the ovary and the size of the nutlet-bearing surface. This system divides the family into a number of subfamilies, clades and subclades. In addition to traditional morphological taxonomy, some of these species have been studied by molecular technologies [14,15,16,17,18]. However, there are significant differences in the results between classical taxonomy and molecular techniques of phylogenetic studies. Different molecular techniques have also given contradictory conclusions to each other. Electrochemical fingerprinting has been shown to be useful in phylogenetic studies at the level of the genus. Exploring the value of the application of this technology in Family level is the aim of this work. In this work, we collected 31 species from 22 genera in the family Lamiaceae and 5 exotaxa as samples to explore the feasibility of fingerprinting recorded by electrochemical sensors in studying the phylogenetic status among genera.

## 2. Materials and Methods

Leaves of *Agastache rugosa*, *Ajuga multiflora*, *Calamintha debilis*, *Galeobdolon chinense*, *Isodon nervosus*, *Lamium barbatum*, *Leonurus japonicus*, *Lycopus lucidus*, *Mentha canadensis*, *Mentha crispate*, *Mentha spicata*, *Mentha vagans*, *Monarda didyma*, *Ocimum basilicum*, *Origanum vulgare*, *Perilla frutescens*, *Physostegia virginiana*, *Prunella vulgaris*, *Salvia elegans*, *Salvia leucantha*, *Salvia miltiorrhiza*, *Salvia splendens*, *Salvia meiliensis*, *Salvia uliginosa*, *Salvia cavaleriei*, *Stachys japonica*, *Elsholtzia cyprianii*, *Hyptis suaveolens*, *Rosmarinus officinalis*, *Vitex negundo*, *Buddleja lindleyana*, *Scrophularia ningpoensis*, *Peristrophe japonica*, *Asystasiella neesiana* and *Nepeta cataria* were supplied by Nanjing Botanic Garden. All fresh leaves were collected from March to July 2021. When collecting, only mature and healthy leaves were harvested. All reagents were analytical grade and used without further purification. 

The extraction process was conducted using ethanol or water as solvent. For a typical extraction process, 1 g of leaves were added into 2 mL of solvent. Then, 2 mill beads were added and the mixture was sonicated at a high throughput tissue grinding machine (MB-24S, Meibi Co Ltd., Hangzhou, China). The extracts after grinding were taken from the supernatant after resting. PBS (pH 7.0) and ABS (pH 4.5) were used as electrolyte. All electrochemical fingerprint recordings were conducted using a CHI760 electrochemical workstation. A three-electrode system has been used for electrochemical fingerprint recording, where a commercial glassy carbon electrode (GCE, 3 mm), an Ag/AgCl electrode and a Pt electrode were used as the working electrode, reference electrode and counter electrode, respectively. For a typical electrochemical fingerprint recording process, 1 mL of plant extract was injected into a 4 mL electrolyte. Then, a differential pulse voltammetry (DPV) was recorded from 0 to 1.3 V. The experimental data was then normalized for further analysis. Principal component analysis (PCA) and cluster analysis were carried out using Origin software with its build-in function. 

## 3. Results and Discussion

**Figure 1:**
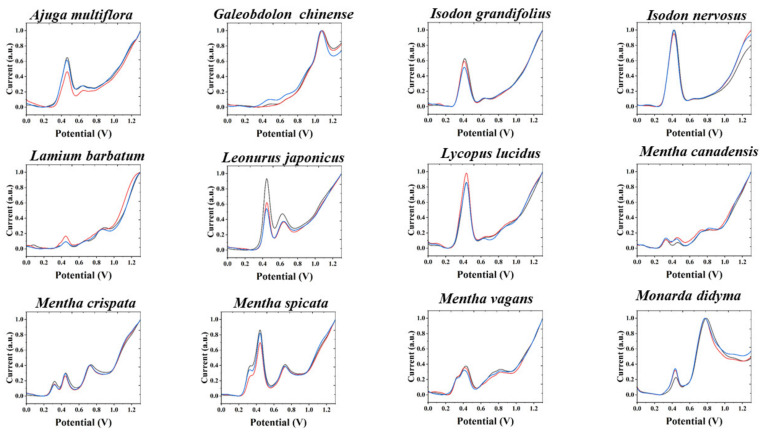
Electrochemical fingerprint of 12 species of Lamiaceae after water extraction and recorded under PBS condition (the remaining 24 species can be found in the Supplementary Information).

Electrochemical fingerprinting is a technique used to record the profile of electrochemical substances in a sample. There is a positive correlation between the difference in electrochemical signals and the type and amount of electrochemically active substances. Therefore, this technique can be used for the identification of complex samples. The electrochemical fingerprints of all plant species under PBS after extraction with water are shown in Figure 1, Figures S1 and S2. From the figures, it can be seen that the electrochemical fingerprints of the plants have very good reproducibility and the three tests basically demonstrate very consistent profiles. According to previous studies, these molecules are mainly flavanols, phenolic acids, procyanidins, alkaloids and pigments. According to our previous studies on electroanalytical chemistry and phytochemistry, the substances that undergo electrochemical oxidation around 0.4 V are most likely ascorbic acid and luteolin. There are many possibilities for other oxidation peaks above 0.6 V. In our experience, substances that can be identified include catechin and coumarin. The electrochemical fingerprints of some of the species have some differences in the peak intensity, but there is no shift in the peak potentials. These differences in peak intensity may be caused by the small electrode area differences generated by different GCEs during polishing. In addition, this could be caused by reasonable differences in the electrochemically active substances in different plant leaves. Plant leaves can have certain differences in their chemical composition because of the different light areas received [19], the different heights of growth [20], etc. It can be seen from the electrochemical fingerprint that all species exhibited oxidation peaks in the anodic scan, which was caused by the oxidation of some electrochemically active substances, such as flavonoids and polyphenols [21,22], in leaf tissues. In contrast to our previous fingerprint profiles of different species within a genus, this time the fingerprint profiles of species within different genera exhibited greater variability. This variability is due to there being less similarity in chemical compositions of the plants between the different genera [23]. However, we can still observe similar fingerprint profiles among some of these species, such as *Isodon grandifolius* vs. *Lycopus lucidus*; *Perilla frutescens* vs. *Salvia meiliensis*; *Salvia splendens* vs. *Peristrophe japonica*. This does not necessarily mean that the leaves of these species contain very similar chemical components, but only that the aqueous extracts of these species have similar electrochemically active substances involved in the oxidation under PBS.

**Figure 2:**
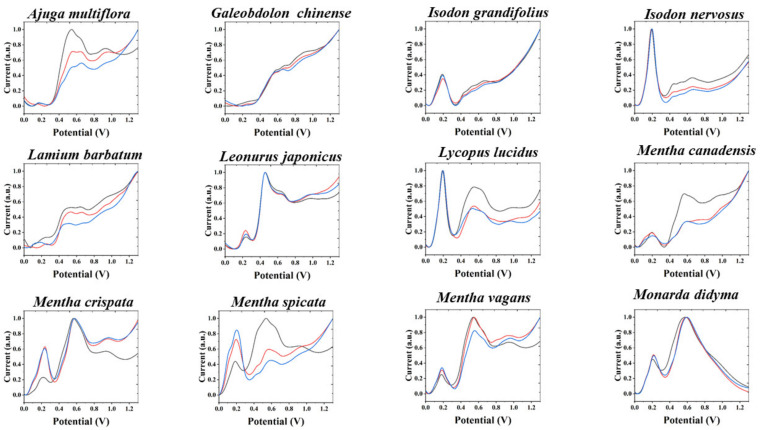
Electrochemical fingerprint of 12 species of Lamiaceae after ethanol extraction and recorded under ABS condition (the remaining 24 species can be found in the Supplementary Information).

Although there are some differences between these similar fingerprints, for example, *Lycopus lucidus* has a relatively small oxidation peak near 0.9 V and *Isodon grandifolius* does not. *Perilla frutescens* has a very large oxidation band between 0.8 and 1.0 V, but *Salvia meiliensis* has two relatively small oxidation peaks. However, using these small differences to distinguish different species is not a very effective method. Therefore, we chose to perform multidimensional fingerprinting of these species. As shown in Figure 2, Figures S3 and S4, we recorded the electrochemical fingerprints of these species under ABS after ethanol extraction. It can be seen that *Isodon grandifolius* and *Lycopus lucidus* exhibit completely different fingerprint profiles. This is because the ethanol extracts of these two species are very different in terms of the substances involved in the electrochemical oxidation under ABS. The same can be observed in *Perilla frutescens* and *Salvia meiliensis*, as well as in *Salvia splendens* and *Peristrophe japonica*. Therefore, multidimensional fingerprinting of plant tissues can increase the abundance of fingerprints and thus improve the correspondence between fingerprints and species. At the same time, multidimensional fingerprinting represents a more comprehensive collection of electrochemical information. For example, more electrochemically active substances can be extracted using polar and non-polar solvents. The use of electrolytes with different pHs also allows more electrochemically active substances to participate in electrochemical reactions.

**Figure 3:**
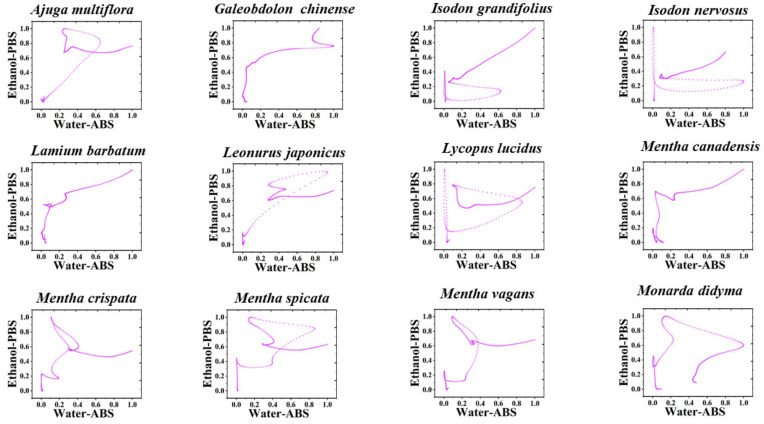
Scatter plots of 12 species of Lamiaceae combining the signals collected under PBS for the water extracts and under ABS for the ethanol extracts (the remaining 24 species can be found in the Supplementary Information).

To further enhance the variability of multidimensional fingerprint profiles in species identification, we deleted the potential information in the fingerprint profiles. Therefore, we can make a scatter plot with the electrochemical fingerprint profiles collected under two different conditions [24,25]. Figure 3, Figures S5 and S6 show scatter plots of *Agastache rugosa*, *Ajuga multiflora*, *Calamintha debilis*, *Galeobdolon chinense*, *Isodon nervosus*, *Lamium barbatum*, *Leonurus japonicus Lycopus lucidus*, *Mentha cana-densis*, *Mentha crispate*, *Mentha spicata*, *Mentha vagans*, *Monarda didyma*, *Ocimum basilicum*, *Origanum vulgare*, *Perilla frutescens*, *Physostegia virginiana*, *Prunella vulgaris*, *Salvia elegans*, *Salvia leucantha*, *Salvia miltiorrhiza*, *Salvia splendens*, *Salvia meiliensis*, *Salvia uliginosa*, *Salvia cavaleriei*, *Stachys japonica*, *Elsholtzia cyprianii*, *Hyptis suaveolens*, *Rosmarinus officinalis*, *Vitex negundo*, *Buddleja lindleyana*, *Scrophularia ningpoensis*, *Peristrophe japonica*, *Asystasiella neesiana* and *Nepeta cataria*. It can be seen from the figure that hardly any two species have very similar scatter plots. Since the points closer to the distal end of the X and Y axes indicate that the oxidation peaks in the original fingerprint profiles, the positioning of the data in the upper right corner of the scatter plot is reflective of the differences between the different species.

**Figure 4:**
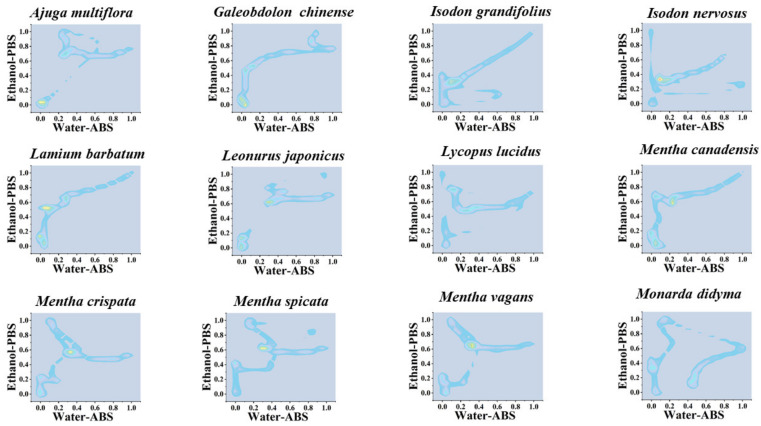
Two-dimensional density map of 12 species of Lamiaceae combining the signals collected under PBS for the water extracts and under ABS for the ethanol extracts (The remaining 24 species can be found in Supplementary Information).

Similarly, the localization of the density of data points can be used for the identification of different species and is more effective than scatter plots. Figure 4, Figures S7 and S8 show 2D density maps of *Agastache rugosa*, *Ajuga multiflora*, *Calamintha debilis*, *Galeobdo-lon chinense*, *Isodon nervosus*, *Lamium barbatum*, *Leonurus japonicus Lycopus lucidus*, *Mentha cana-densis*, *Mentha crispate*, *Mentha spicata*, *Mentha vagans*, *Monarda didyma*, *Ocimum basili-cum*, *Origanum vulgare Perilla frutescens*, *Physostegia virginiana*, *Prunella vulgaris*, *Salvia ele-gans*, *Salvia leucantha*, *Salvia miltiorrhiza*, *Salvia splendens*, *Salvia uliginensis meiliensis*, *Salvia uliginosa*, *Salvia cavaleriei*, *Stachys japonica*, *Elsholtzia cyprianii*, *Hyptis suaveolens*, *Rosmarinus officinalis*, *Vitex negundo*, *Buddleja lindleyana*, *Scrophularia ningpoensis*, *Peristrophe japonica*, *Asystasiella neesiana and Nepeta cataria*. In the 2D density maps, the data-dense regions are highlighted. These highlighted areas represent that different fingerprints on the same interval of potential all show similar electrochemical correspondence. At the same time, some points with more dispersed data are weakened in the 2D density maps. Therefore, only the highlighted areas need to be located to correlate to the corresponding species.

**Figure 5:**
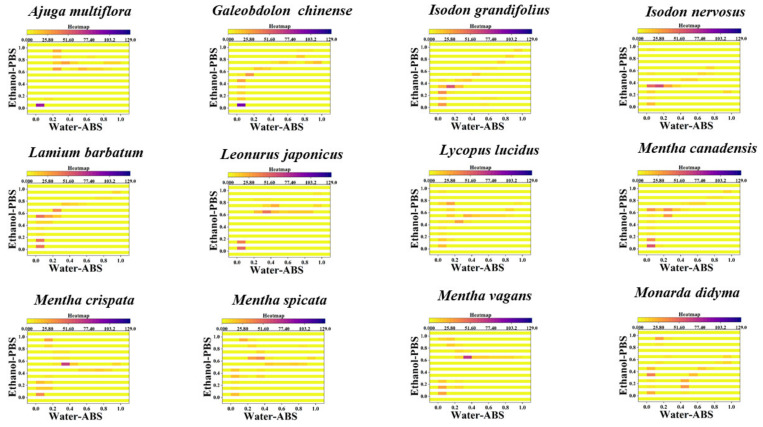
Heatmap of 12 species of Lamiaceae combining the signals collected under PBS for the water extracts and under ABS for the ethanol extracts (the remaining 24 species can be found in the Supplementary Information).

Heatmaps can improve the accuracy of pattern recognition even further based on 2D density maps. Figure 5, Figure S9 and S10 show heatmap of *Agastache rugosa*, *Ajuga multiflora*, *Calamintha debilis*, *Galeobdo-lon chinense*, *Isodon nervosus*, *Lamium barbatum*, *Leonurus japonicus Lycopus lucidus*, *Mentha cana-densis*, *Mentha crispate*, *Mentha spicata*, *Mentha vagans*, *Monarda didyma*, *Ocimum basili-cum*, *Origanum vulgare Perilla frutescens*, *Physostegia virginiana*, *Prunella vulgaris*, *Salvia ele-gans*, *Salvia leucantha*, *Salvia miltiorrhiza*, *Salvia splendens*, *Salvia uliginensis meiliensis*, *Salvia uliginosa*, *Salvia cavaleriei*, *Stachys japonica*, *Elsholtzia cyprianii*, *Hyptis suaveolens*, *Rosmarinus officinalis*, *Vitex negundo*, *Buddleja lindleyana*, *Scrophularia ningpoensis*, *Peristrophe japonica*, *Asystasiella neesiana and Nepeta cataria*. As can be seen in the figure, the heatmap has the whole graph segmented in addition to the highlighted regions. Therefore, in this case, in addition to locating hot areas, counting the number of hot areas can be used for the identification of different species. Overall, these previously established pattern recognition techniques can be used well for the identification of species in Lamiaceae. It can be seen from the different output patterns that the differences in the pattern of plants under different genera are greater than the previous differences between different species under the same genus [26,27,28,29]. This also corresponds to the pattern of biological evolution. Although in the evolution of organisms, plants with more distant phylogenetic status possess less similar chemical composition to each other. This is because the number of phytochemical species depends mainly on the expression of genes. Large differences in gene expression can lead to large differences between phytochemical components and, in turn, to large differences between fingerprint profiles [30,31].

**Figure 6:**
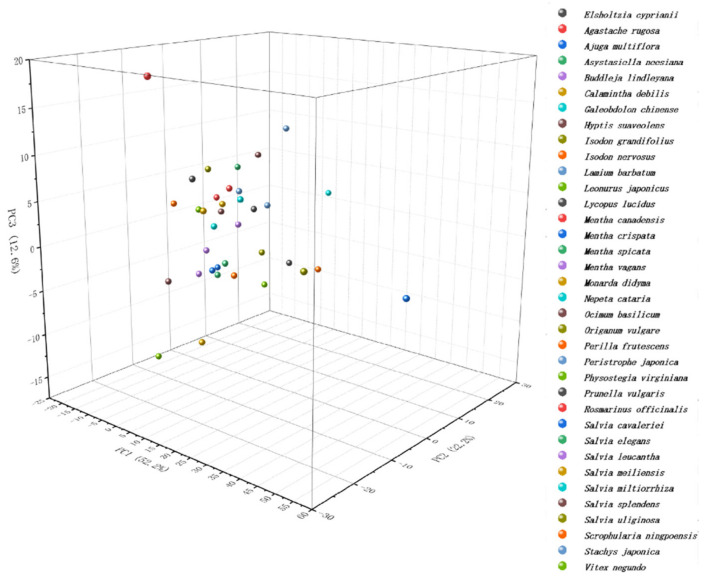
PCA analysis of 31 species from 22 genera in the family Lamiaceae and 5 exotaxa in this work.

Phytochemical taxonomy has always been a methodological approach in plant taxonomy [32]. The basis of taxonomic study of plants using their compositional differences is that the compositional differences of plants reflect their differences at the genetic level [33,34]. However, phytochemical taxonomy has its limitations because qualitative and quantitative analysis of a large number of phytochemical components is very difficult. Therefore, previous studies have tended to track only some secondary metabolites of plants as markers [35,36]. These markers are often chosen to study the more specific components of the species, but there is no particularly logical reason for this. Fingerprinting of plants with electrochemical biosensors can present a large amount of overall information about electrochemically active substances, so this can be used as a kind of big data for analyzing the variability of all electrochemically active substances in different plants, and therefore has the potential to be applied to phylogenetic studies of plants. We first overlaid and normalized the multidimensional fingerprint profiles of all species, and then performed PCA on these data. Figure 6 shows PCA of *Agastache rugosa*, *Ajuga multiflora*, *Calamintha debilis*, *Galeobdolon chinense*, *Isodon nervosus*, *Lamium barbatum*, *Leonurus japonicus*, *Lycopus lucidus*, *Mentha canadensis*, *Mentha crispate*, *Mentha spicata*, *Mentha vagans*, *Monarda didyma*, *Ocimum basilicum*, *Origa-num vulgare*, *Perilla frutescens*, *Physostegia virginiana*, *Prunella vulgaris*, *Salvia elegans*, *Salvia leucantha*, *Salvia miltiorrhiza*, *Salvia splendens*, *Salvia meiliensis*, *Salvia uliginosa*, *Salvia cava-leriei*, *Stachys japonica*, *Elsholtzia cyprianii*, *Hyptis suaveolens*, *Rosmarinus officinalis*, *Vitex negundo*, *Buddleja lindleyana*, *Scrophularia ningpoensis*, *Peristrophe japonica*, *Asystasiella neesiana* and *Nepeta cataria*. After extracting three factors, PCA could reach 87% interpretation, indicating the electrochemical fingerprint contains representative information that can be used to represent different data sets. Since here we use 3D PCA, the distance between different species is difficult to measure. The distances between different species will be presented with dendrogram.

**Figure 7:**
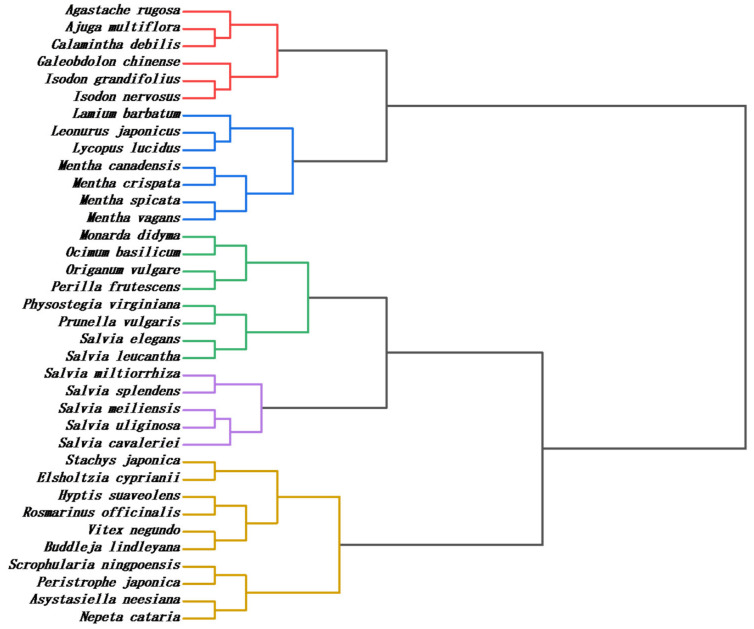
Dendrogram of 31 species from 22 genera in the family Lamiaceae and 5 exotaxa based on electrochemical fingerprints.

The entire phylogenetic tree is divided into five main clades (Figure 7). The first clade contains *Agastache rugosa*, *Ajuga multiflora*, *Calamintha debilis*, *Galeobdolon chinense*, *Isodon grandifolous* and *Isodon nervosus*. The second clade contains *Lamium barbatum*, *Leonurus japonicus*, *Lycopus lucidus*, *Mentha canadensis*, *Mentha crispate*, *Mentha spicata* and *Mentha vagans*. The third clade contains *Monarda didyma*, *Ocimum basilicum*, *Origanum vulgare*, *Perilla frutescens*, *Physostegia virginiana*, *Prunella vulgaris*, *Salvia elegans* and *Salvia leucantha*. The fourth clade contains *Salvia miltiorrhiza*, *Salvia splendens*, *Salvia meiliensis*, *Salvia uliginosa*, *and Salvia cavaleriei*. The fifth clade *contains Stachys japonica*, *Elsholtzia cyprianii*, *Hyptis suaveolens*, *Rosmarinus officinalis*, *Vitex negundo*, *Buddleja lindleyana*, *Scrophularia ningpoensis*, *Peristrophe japonica*, *Asystasiella neesiana* and *Nepeta cataria*.

Surprisingly, all five exotaxa were clustered in the last cluster. The distant affinity of *Vitex negundo* and *Nepeta cataria* from the Lamiaceae can be confirmed by recent molecular study. Ayaz et al. [37] isolated molecular DNA from fresh leaf. The rps14 gene was amplified for the isolation of DNA sequencing and consequently used for phylogenetic analysis. Both *Vitex negundo* and *Nepeta cataria* showed genetically different from the rest of species from Lamiaceae. The close relationship between *Buddleja lindleyana* with genus *Scrophularia* was confirmed by the recent complete chloroplast genome analysis [38]. Similarly, the complete chloroplast genome analysis of *Peristrophe japonica* indicated its close relationship with *Scrophularia* and *Asystasiella* [39].

All the plants of the genus *Mentha* were clustered together, which fits well with the results of either classical morphological classification or molecular studies. Since the genus *Mentha* was established in 1753 by the Linnaeus, scholars have conducted extensive research on the genus in terms of morphology, cytology, physiology, biochemistry, phytochemistry and molecular biology. The chemical classification of *Mentha* is mainly based on monoterpene components. Pulotova [40] classified this genus into three groups: carvone group, menthone group, and mixed group. Later, Tucker et al. [41] also studied the chemical classification of the *Mentha* [19]. Murray and Lincoln [42] pointed out that oxygen-containing p-menthane-type monoterpenes are chemically characteristic of the *Mentha*. Since species of the *Mentha* share a greater similarity in chemical composition, they also possess minor differences in electrochemical fingerprinting and are thus clustered together. Khanuja et al. had analyzed the genetic relationships of 11 mint plants using RAPD technique with 60 random primers [43]. Both *Mentha arvensis* and *Mentha spicata* were highly correlated by RAPD analysis. This is consistent with the conclusion we proposed. *Isodon grandifolius* and *Isodon nervosus* were clustered together, which is also consistent with previous molecular phylogenetic studies [44,45,46,47].

Most species of *Salvia* were clustered together in the same clade. The close relationship between *Salvia meiliensis* and *Salvia miltiorrhiza* can be confirmed by the chloroplast genome analysis, and they all belong to sub-genus *Glutinaria* [48]. However, the position of *Salvia splendens* did not exactly correspond to the results of the previous chloroplast genome analysis. Although the chloroplast genome analysis showed the close relationship of *Salvia splendens* to sub-genus *Glutinaria*, it belongs to sub-genus *Calosphace*, while its phylogenetic status showed a very close relationship to *Salvia miltiorrhiza* in our result. Since the remaining *Salvia* species were not included in the previously published molecular studies, our results could provide a direction for future studies. The complete chloroplast genomes analysis of *Lycopus lucidus* and *Agastache rugosa* showed they belong to tribe Mentheae of Lamiaceae family [49]. Our results give strong support to this argument.

## 4. Conclusions

In conclusion, this work attempts to use fingerprinting collected with electrochemical biosensors for the identification and phylogenetic study of species in Lamiaceae. The results demonstrate that electrochemical fingerprinting is more effective in the identification of species of a family than previously in the identification of species of a genus. This observation is due to the fact that the phytochemical composition of species differs much more between genera than those in the same genus. In phylogenetic studies, electrochemical fingerprinting shows the potential to be studied among different genera.

## Data Availability

Data sharing not applicable.

## Supplementary Materials

The following are available online at https://www.mdpi.com/article/10.3390/s21248216/s1, Figure S1. Electrochemical fingerprint of 12 species of Lamiaceae after water extraction and recorded under PBS condition (the remaining 24 species can be found in the Supplementary Information), Figure S2. Electrochemical fingerprint of 12 species of Lamiaceae after ethanol extraction and recorded under ABS condition (the remaining 24 species can be found in the Supplementary Information), Figure S3. Scatter plots of 12 species of Lamiaceae combining the signals collected under PBS for the water extracts and under ABS for the ethanol extracts (the remaining 24 species can be found in the Supplementary Information), Figure S4. Two-dimensional density map of 12 species of Lamiaceae combining the signals collected under PBS for the water ex-tracts and under ABS for the ethanol extracts (The remaining 24 species can be found in Supplementary Information), Figure S5. Heatmap of 12 species of Lamiaceae combining the signals collected under PBS for the water extracts and under ABS for the ethanol extracts (the remaining 24 species can be found in the Supplementary Information), Figure S6. PCA analysis of 31 species from 22 genera in the family Lamiaceae and 5 exotaxa in this work, Figure S7. Dendrogram of 31 species from 22 genera in the family Lamiaceae and 5 exotaxa based on electrochemical fingerprints.

## References

[1] Doménech-Carbó A., Doménech-Carbó M.T., Ferragud-Adam X., Ortiz-Miranda A.S., Montoya N., Pasíes-Oviedo T., Peiró-Ronda M.A., Vives-Ferrándiz J., Marco Y.C. (2017). Identification of Vegetal Species in Wooden Objects Using in Situ Microextrac-tion-Assisted Voltammetry of Microparticles. Anal. Methods.

[2] Fan B., Wang Q., Wu W., Zhou Q., Li D., Xu Z., Fu L., Zhu J., Karimi-Maleh H., Lin C.-T. (2021). Electrochemical Fingerprint Biosensor for Natural Indigo Dye Yielding Plants Analysis. Biosensors.

[3] Fu L., Zheng Y., Zhang P., Zhang H., Zhuang W., Zhang H., Wang A., Su W., Yu J., Lin C.-T. (2018). Enhanced Electrochemical Voltammetric Fingerprints for Plant Taxonomic Sensing. Biosens. Bioelectron..

[4] Doménech-Carbó A., Ramírez-Barat B., Petiti C., Goidanich S., Doménech-Carbó M.T., Cano E. (2020). Characterization of traditional artificial patinas on copper using the voltammetry of immobilized particles. J. Electroanal. Chem..

[5] Doménech-Carbó A., Donnici M., Álvarez-Romero C., Daniele S., Doménech-Carbó M.T. (2021). Multiple-scan voltammetry of immobilized particles of ancient copper/bronze coins. J. Solid State Electrochem..

[6] Novak I., Mlakar M., Komorsky-Lovrić Š. (2013). Voltammetry of Immobilized Particles of Cannabinoids. Electroanalysis.

[7] Zhou J., Zheng Y., Zhang J., Karimi-Maleh H., Xu Y., Zhou Q., Fu L., Wu W. (2020). Characterization of the Electrochemical Profiles of Lycoris Seeds for Species Identification and Infrageneric Relationships. Anal. Lett..

[8] Wang Y., Pan B., Zhang M., Du X., Wu W., Fu L., Zhou W., Zheng Y. (2020). Electrochemical Profile Recording for Pueraria Variety Identification. Anal. Sci..

[9] Xu Y., Lu Y., Zhang P., Wang Y., Zheng Y., Fu L., Zhang H., Lin C.-T., Yu A. (2020). Infrageneric phylogenetics investigation of Chimonanthus based on electroactive compound profiles. Bioelectrochemistry.

[10] Zheng Y., Wang D., Li X., Wang Z., Zhou Q., Fu L., Yin Y., Creech D. (2021). Biometric Identification of *Taxodium* spp. and Their Hybrid Progenies by Electrochemical Fingerprints. Biosensors.

[11] Karimi-Maleh H., Khataee A., Karimi F., Baghayeri M., Fu L., Rouhi J., Karaman C., Karaman O., Boukherroub R. (2021). A Green and Sensitive Guanine-Based DNA Biosensor for Idarubicin Anticancer Monitoring in Biological Samples: A Simple and Fast Strategy for Control of Health Quality in Chemotherapy Procedure Confirmed by Docking Investigation. Chemosphere.

[12] Karpiński T.M. (2020). Essential Oils of Lamiaceae Family Plants as Antifungals. Biomolecules..

[13] Briquet J.-L., Sawicki F. (1989). L’analyse Localisée Du Politique. Politix. The localized analysis of politics.

[14] Zimowska B., Okoń S., Becchimanzi A., Krol E.D., Nicoletti R. (2020). Phylogenetic Characterization of Botryosphaeria Strains Associated with Asphondylia Galls on Species of Lamiaceae. Divers.

[15] Bunsawat J., Elliott N.E., Hertweck K.L., Sproles E., Alice L.A. (2004). Phylogenetics of Mentha (Lamiaceae): Evidence from Chloroplast DNA Sequences. Syst. Bot..

[16] Drew B.T., Sytsma K.J. (2012). Phylogenetics, biogeography, and staminal evolution in the tribe Mentheae (Lamiaceae). Am. J. Bot..

[17] Chen Y.-P., Drew B.T., Li B., Soltis D.E., Soltis P.S., Xiang C.-L. (2016). Resolving the Phylogenetic Position of Ombrocharis (La-miaceae), with Reference to the Molecular Phylogeny of Tribe Elsholtzieae. Taxon.

[18] Drew B.T., Sytsma K.J. (2013). The South American radiation of Lepechinia (Lamiaceae): Phylogenetics, divergence times and evolution of dioecy. Bot. J. Linn. Soc..

[19] Grauso L., de Falco B., Motti R., Lanzotti V. (2021). Corn poppy, Papaver rhoeas L.: A critical review of its botany, phytochemistry and pharmacology. Phytochem. Rev..

[20] Wakawa A.I., Audu B., Sulaiman Y. (2018). Phytochemistry and Proximate Composition of Root, Stem Bark, Leaf and Fruit of Desert Date, Balanites Aegyptiaca. J. Phytopharm..

[21] Geng R., Ma L., Liu L., Xie Y. (2018). Influence of Bovine Serum Albumin-Flavonoid Interaction on the Antioxidant Activity of Dietary Flavonoids: New Evidence from Electrochemical Quantification. Molecules.

[22] Chiorcea-Paquim A., Enache T.A., Gil E.D.S., Oliveira-Brett A.M. (2020). Natural phenolic antioxidants electrochemistry: Towards a new food science methodology. Compr. Rev. Food Sci. Food Saf..

[23] Zhang B.-M., Zhi-Bin W., Ping X., Qiu-Hong W., He B., Kuang H.-X. (2018). Phytochemistry and Pharmacology of Genus Ephedra. Chin. J. Nat. Med..

[24] Fu L., Su W., Chen F., Zhao S., Zhang H., Karimi-Maleh H., Yu A., Yu J., Lin C.-T. (2021). Early sex determination of Ginkgo biloba based on the differences in the electrocatalytic performance of extracted peroxidase. Bioelectrochemistry.

[25] Zhang X., Yang R., Li Z., Zhang M., Wang Q., Xu Y., Fu L., Du J., Zheng Y., Zhu J. (2020). Electroanalytical Study of Infrage-neric Relationship of Lagerstroemia Using Glassy Carbon Electrode Recorded Voltammograms. Rev. Mex. Ing. Quím..

[26] Fu L., Wang Q., Zhang M., Zheng Y., Wu M., Lan Z., Pu J., Zhang H., Chen F., Su W. (2020). Electrochemical Sex Determina-tion of Dioecious Plants Using Polydopamine-Functionalized Graphene Sheets. Front. Chem..

[27] Yang R., Fan B., Wang S., Li L., Li Y., Li S., Zheng Y., Fu L., Lin C.-T. (2020). Electrochemical Voltammogram Recording for Identifying Varieties of Ornamental Plants. Micromachines.

[28] Fu L., Zheng Y., Zhang P., Zhu J., Zhang H., Zhang L., Su W. (2018). Embedding Leaf Tissue in Graphene Ink to Improve Signals in Electrochemistry-Based Chemotaxonomy. Electrochem. Commun..

[29] Fu L., Zheng Y., Wang A., Zhang P., Ding S., Wu W., Zhou Q., Chen F., Zhao S. (2021). Identification of medicinal herbs in Asteraceae and Polygonaceae using an electrochemical fingerprint recorded using screen-printed electrode. J. Herb. Med..

[30] Macias F.A., Galindo J.L., Galindo J.C. (2007). Evolution and Current Status of Ecological Phytochemistry. Phytochemistry.

[31] Frezza C., Venditti A., Giuliani C., Foddai S., Cianfaglione K., Maggi F., Fico G., Guiso M., Nicoletti M., Bianco A. (2021). Occurrence of flavonoids in different Lamiaceae taxa for a preliminary study on their evolution based on phytochemistry. Biochem. Syst. Ecol..

[32] Ortiz A.C., Spampinato G., Fuentes J.P., Gomes C.P., Quinto-Canas R., Cano E. (2021). Taxonomy, Ecology and Distribution of *Juniperus oxycedrus* L. Group in the Mediterranean Basin Using Bioclimatic, Phytochemical and Morphometric Approaches, with Special Reference to the Iberian Peninsula. Forests.

[33] Karimi-Maleh H., Karimi F., Fu L., Sanati A.L., Alizadeh M., Karaman C., Orooji Y. (2022). Cyanazine Herbicide Monitoring as a Hazardous Substance by a DNA Nanostructure Biosensor. J. Hazard. Mater..

[34] Thompson H., Levitt J., McGinley J., Chandler P., Guenther P., Huybrechts I., Playdon M. (2021). Measuring Dietary Botanical Diversity as a Proxy for Phytochemical Exposure. Nutrients.

[35] Ullah F., Ayaz A., Saqib S., Zaman W., Butt M.A., Ullah A. (2019). Silene Conoidea L.: A Review on Its Systematic, Ethnobota-ny and Phytochemical Profile. Plant Sci. Today.

[36] Roma-Marzio F., Najar B., Alessandri J., Pistelli L., Peruzzi L. (2017). Taxonomy of prickly juniper (Juniperus oxycedrus group): A phytochemical–morphometric combined approach at the contact zone of two cryptospecies. Phytochemistry.

[37] Ayaz A., Zaman W., Saqib S., Ullah F., Mahmood T. (2020). Phylogeny and diversity of lamiaceae based on rps14 gene in Pakistan. Genetika.

[38] Liu S., Feng S., Huang Y., An W., Yang Z., Xie C., Zheng X. (2021). Characterization of the Complete Chloroplast Genome of Buddleja Lindleyana. J. AOAC Int..

[39] Chen J., Wang L., Zhao Y., Qin M. (2021). The complete chloroplast genome of a Chinese medicinal plant, Peristrophe japonica (Thunb.) Bremek. (Lamiales: Acanthaceae) from Nanjing, China. Mitochondrial DNA Part B Resour..

[40] Pulotova T. (1973). Phenolic Compounds of Some Species of Mint. Uzb. Biol. Zh.

[41] Tucker A.O., Hendriks H., Bos R., Fairbrothers D.E. (1991). The Origin of Mentha—Gracilis (Lamiaceae). II. Essential Oils. Econ. Bot..

[42] Murray M., Lincoln D., Marble P. (1972). Oil Composition of Mentha Aquatica x Mentha Spicata F1 Hybrids in Relation to the Origin of x Mentha Piperita. Can. J. Genet. Cytology.

[43] Khanuja S., Shasany A., Srivastava A., Kumar S. (2000). Assessment of Genetic Relationships in Mentha Species. Euphytica.

[44] Chen Y.-P., Wilson T.C., Zhou Y.-D., Wang Z.-H., Liu E.-D., Peng H., Xiang C.-L. (2019). Isodon Hsiwenii (Lamiaceae: Nepe-toideae), a New Species from Yunnan, China. Syst. Bot..

[45] Chen Y.-P., Huang C.-Z., Zhao Y., Xiang C.-L. (2021). Molecular and Morphological Evidence for a New Species of Isodon (La-miaceae) from Southern China. Plant Diversity.

[46] Yu X.-Q., Maki M., Drew B.T., Paton A.J., Li H.-W., Zhao J.-L., Conran J.G., Li J. (2014). Phylogeny and Historical Biogeogra-phy of Isodon (Lamiaceae): Rapid Radiation in South-West China and Miocene Overland Dispersal into Africa. Mol. Phyloge-net. Evol..

[47] Zhong J.-S., Li J., Li L., Conran J.G., Li H.-W. (2010). Phylogeny of Isodon (Schrad. Ex Benth.) Spach (Lamiaceae) and Related Genera Inferred from Nuclear Ribosomal ITS, TrnL–TrnF Region, and Rps16 Intron Sequences and Morphology. Syst. Bot..

[48] Zhao F., Drew B.T., Chen Y.-P., Hu G.-X., Li B., Xiang C.-L. (2020). The Chloroplast Genome of Salvia: Genomic Characteriza-tion and Phylogenetic Analysis. Int. J. Plant Sci..

[49] Wang Y., Wang H., Zhou B., Yue Z. (2021). The Complete Chloroplast Genomes of Lycopus Lucidus and Agastache Rugosa, Two Herbal Species in Tribe Mentheae of Lamiaceae Family. Mitochondrial DNA Part B.

