# Supplementary material for: Can Electrochemical Sensors Be Used for Identification and Phylogenetic Studies in Lamiaceae?
Source: Sensors (Basel). 2021 Dec 8;21(24):8216. doi: 10.3390/s21248216 (PMC8706286; doi:10.3390/s21248216)
Supplement: Supplementary file 1 [file sensors-21-08216-s001.zip › sensors-1466218-supplementary.pdf]

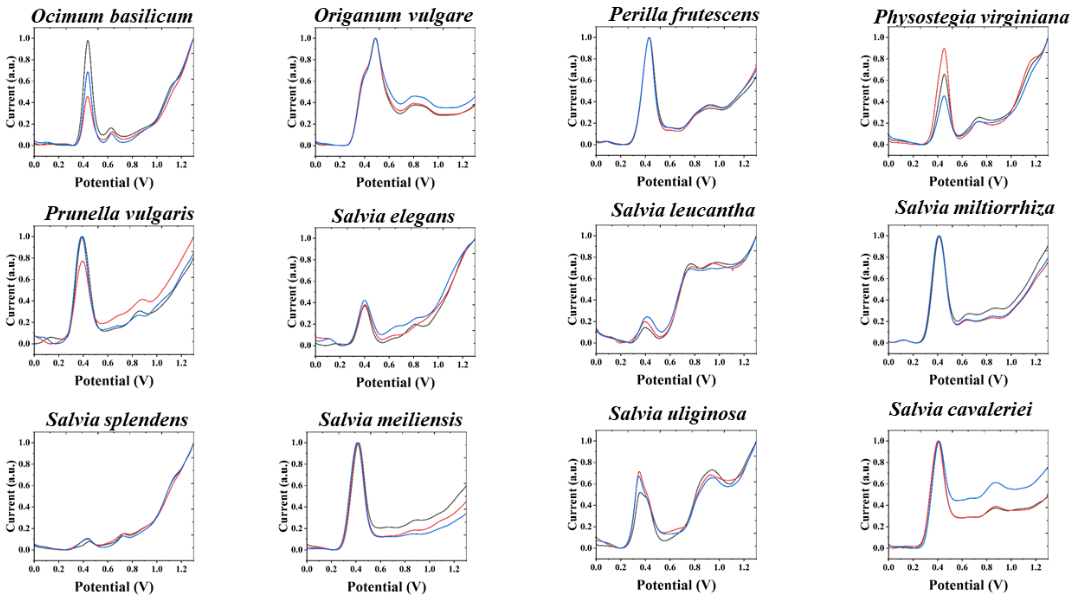

Figure S1. Electrochemical fingerprint of *Ocimum basilicum, Origanum vulgare, Perilla frutescens, Physostegia virginiana, Prunella vulgaris, Salvia elegans, Salvia leucantha, Salvia miltiorrhiza, Salvia splendens, Salvia meiliensis, Salvia uliginosa, Salvia cavaleriei* after water extraction and recorded under PBS condition.

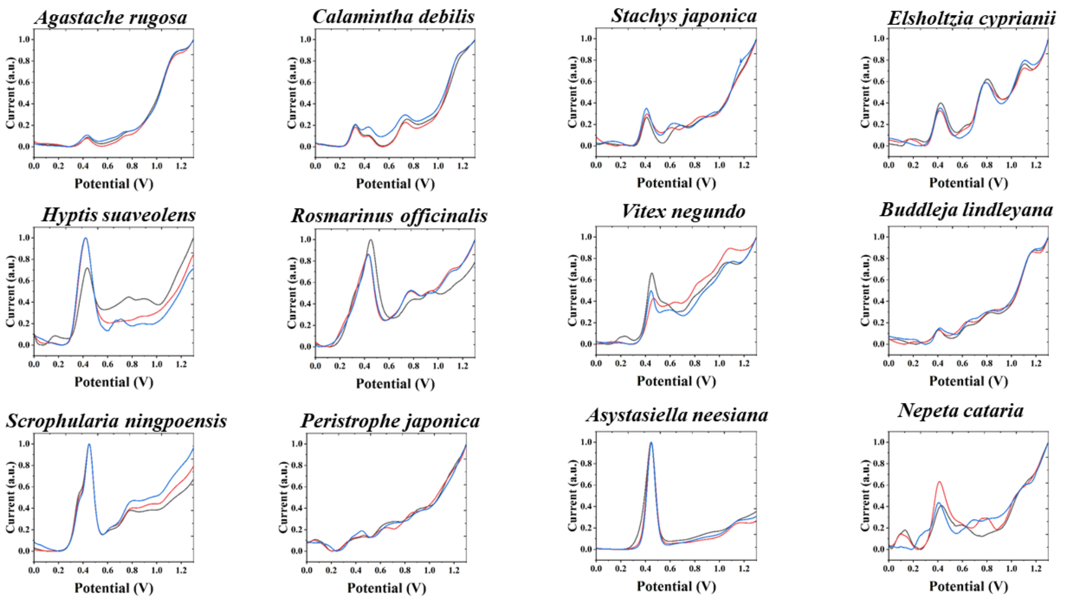

Figure S2. Electrochemical fingerprint of *Agastache rugosa, Calamintha debilis, Stachys japonica, Elsholtzia cyprianii, Hyptis suaveolens, Rosmarinus officinalis, Vitex negundo, Buddleja lindleyana, Scrophularia ningpoensis, Peristrophe japonica, Asystasiella neesiana, Nepeta cataria* after water extraction and recorded under PBS condition.

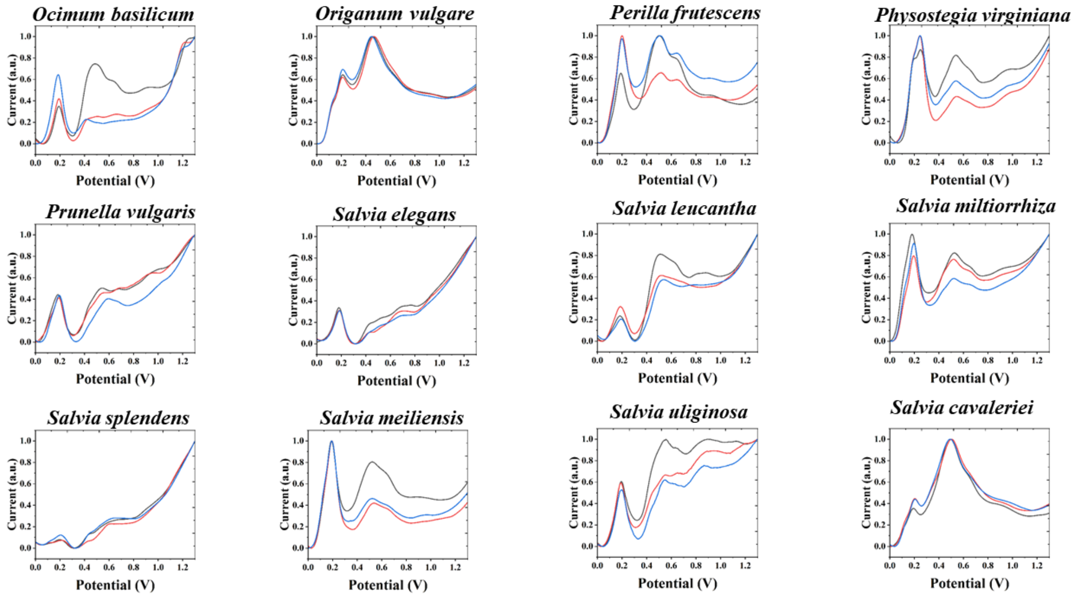

Figure S3. Electrochemical fingerprint of *Ocimum basilicum, Origanum vulgare, Perilla frutescens, Physostegia virginiana, Prunella vulgaris, Salvia elegans, Salvia leucantha, Salvia miltiorrhiza, Salvia splendens, Salvia meiliensis, Salvia uliginosa, Salvia cavaleriei* after ethanol extraction and recorded under ABS condition.

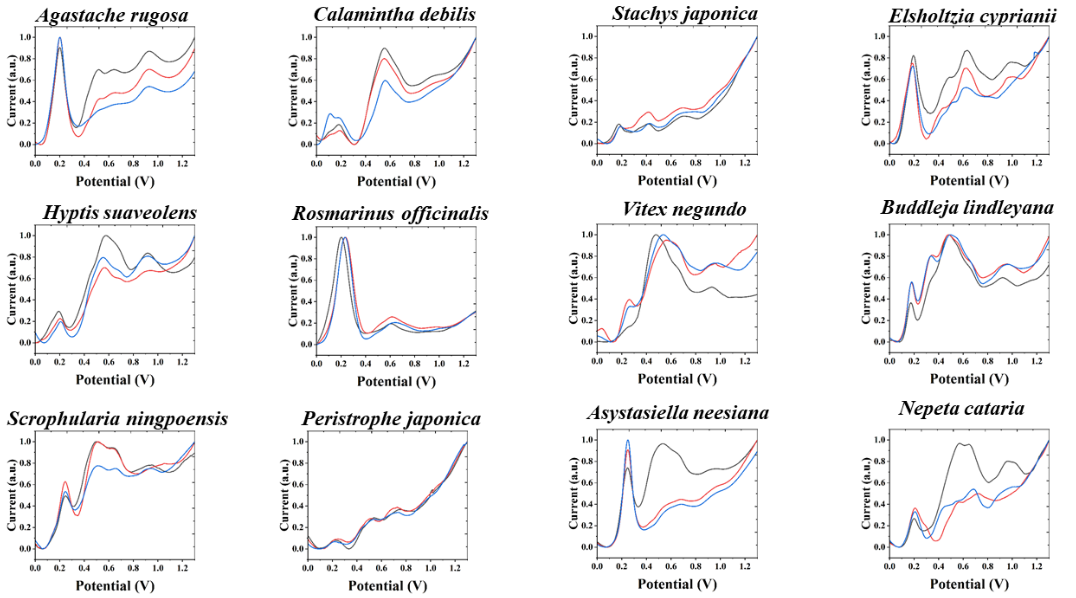

Figure S4. Electrochemical fingerprint of *Agastache rugosa, Calamintha debilis, Stachys japonica, Elsholtzia cyprianii, Hyptis suaveolens, Rosmarinus officinalis, Vitex negundo, Buddleja lindleyana, Scrophularia ningpoensis, Peristrophe japonica, Asystasiella neesiana, Nepeta cataria* after ethanol extraction and recorded under ABS condition.

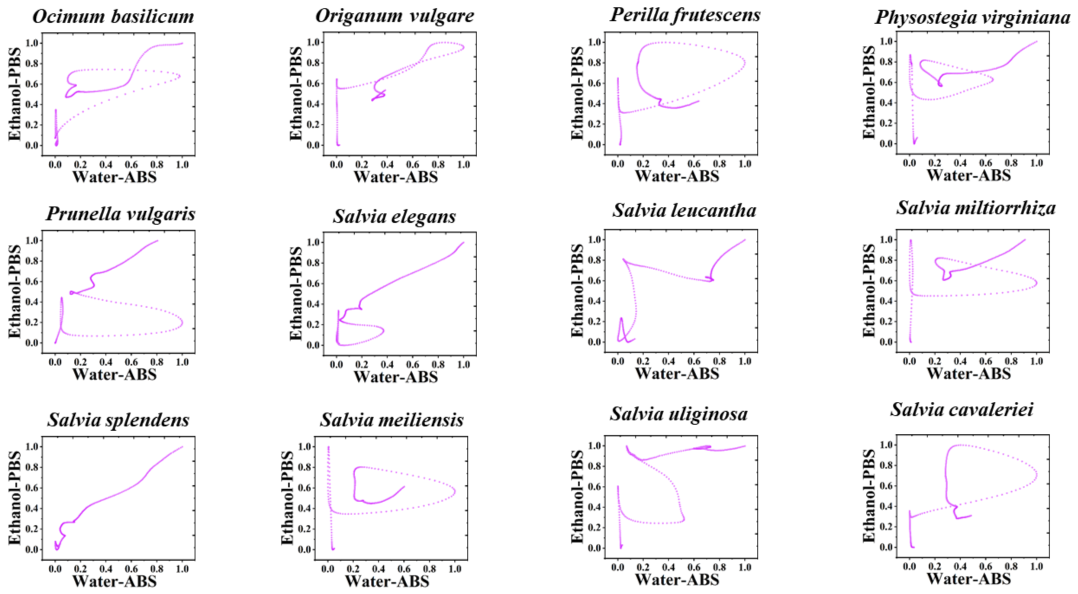

Figure S5. Scatter plots of *Ocimum basilicum, Origanum vulgare, Perilla frutescens, Physostegia virginiana, Prunella vulgaris, Salvia elegans, Salvia leucantha, Salvia miltiorrhiza, Salvia splendens, Salvia meiliensis, Salvia uliginosa, Salvia cavaleriei* combining the signals collected under PBS for the water extracts and under ABS for the ethanol extracts.

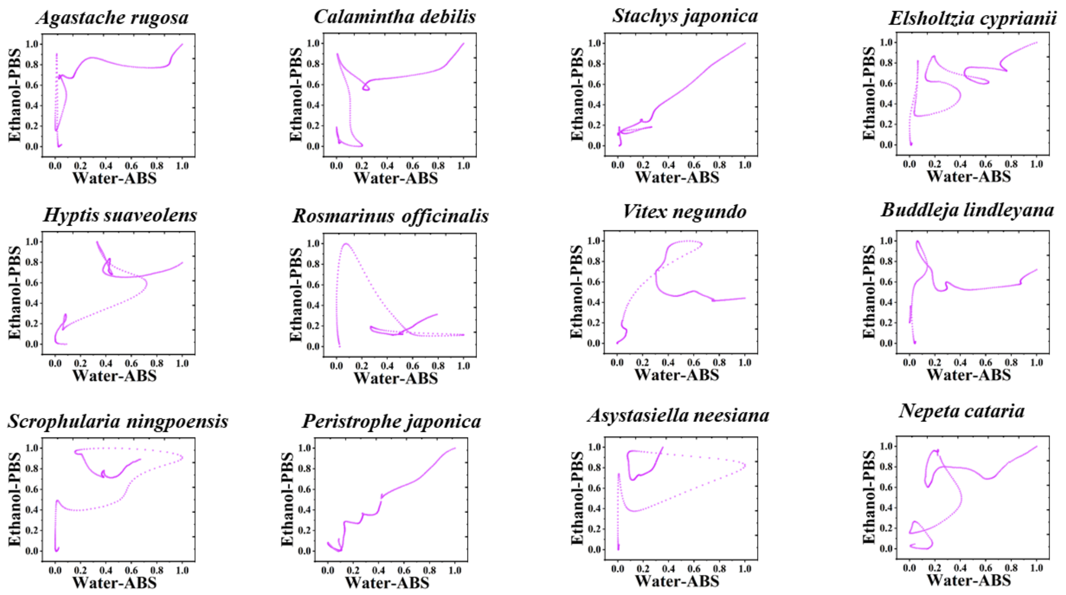

Figure S6. Scatter plots of *Agastache rugosa, Calamintha debilis, Stachys japonica, Elsholtzia cyprianii, Hyptis suaveolens, Rosmarinus officinalis, Vitex negundo, Buddleja lindleyana, Scrophularia ningpoensis, Peristrophe japonica, Asystasiella neesiana, Nepeta cataria* combining the signals collected under PBS for the water extracts and under ABS for the ethanol extracts.

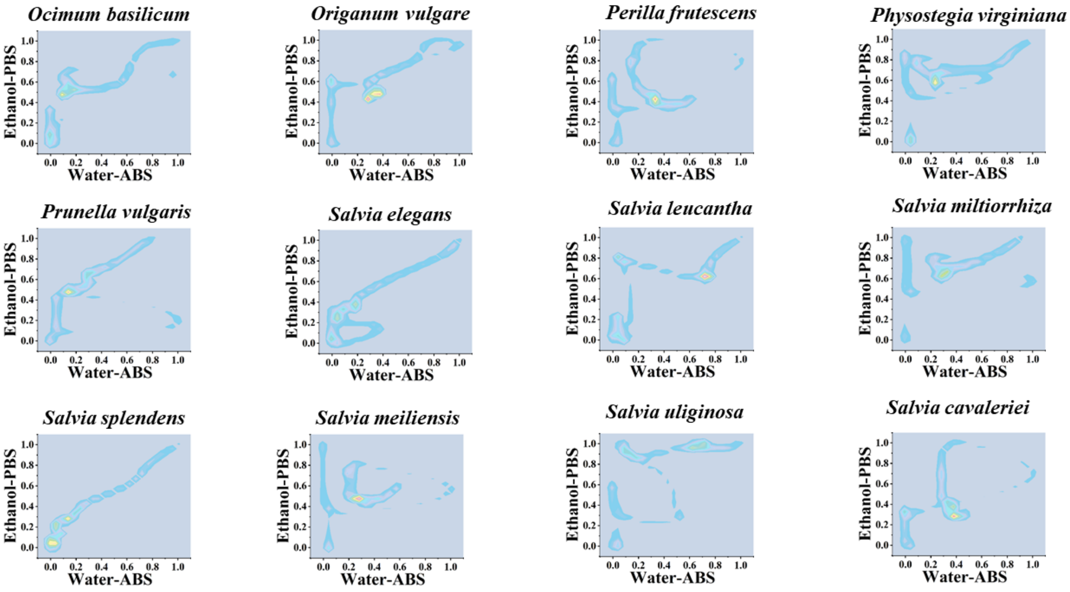

Figure S7. 2D density map of *Ocimum basilicum, Origanum vulgare, Perilla frutescens, Physostegia virginiana, Prunella vulgaris, Salvia elegans, Salvia leucantha, Salvia miltiorrhiza, Salvia splendens, Salvia meiliensis, Salvia uliginosa, Salvia cavaleriei* combining the signals collected under PBS for the water extracts and under ABS for the ethanol extracts.

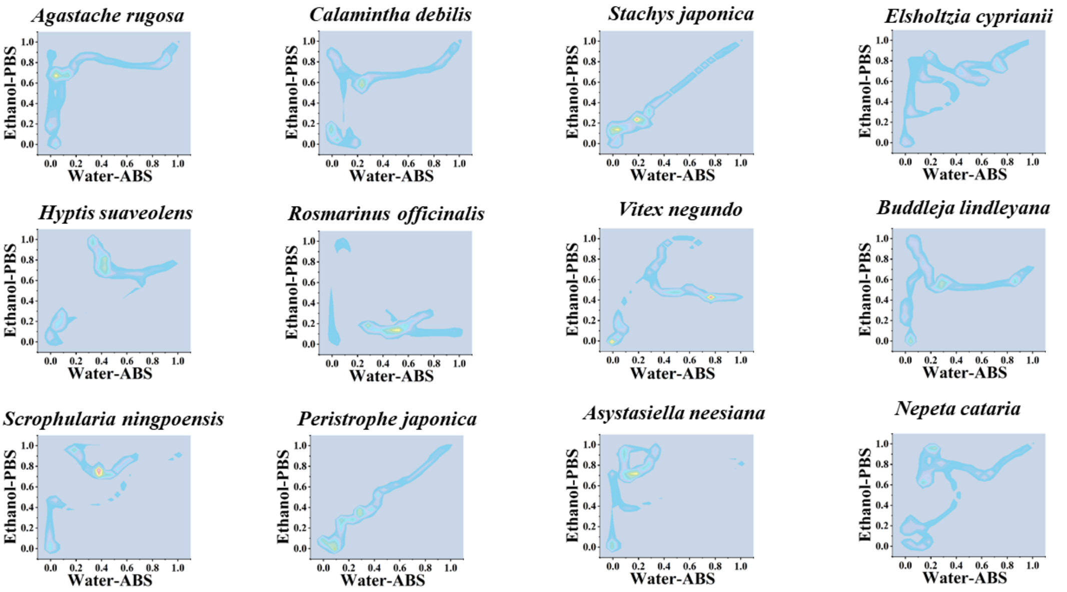

Figure S8. 2D density map of *Agastache rugosa, Calamintha debilis, Stachys japonica, Elsholtzia cyprianii, Hyptis suaveolens, Rosmarinus officinalis, Vitex negundo, Buddleja lindleyana, Scrophularia ningpoensis, Peristrophe japonica, Asystasiella neesiana, Nepeta cataria* combining the signals collected under PBS for the water extracts and under ABS for the ethanol extracts.

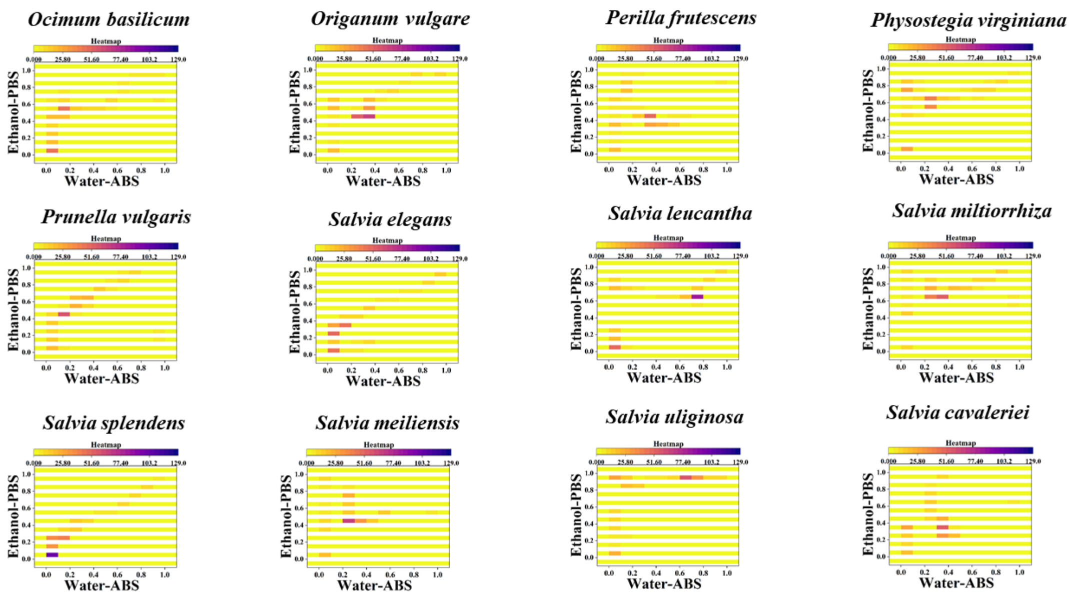

Figure S9. Heatmap of *Ocimum basilicum, Origanum vulgare, Perilla frutescens, Physostegia virginiana, Prunella vulgaris, Salvia elegans, Salvia leucantha, Salvia miltiorrhiza, Salvia splendens, Salvia meiliensis, Salvia uliginosa, Salvia cavaleriei* combining the signals collected under PBS for the water extracts and under ABS for the ethanol extracts.

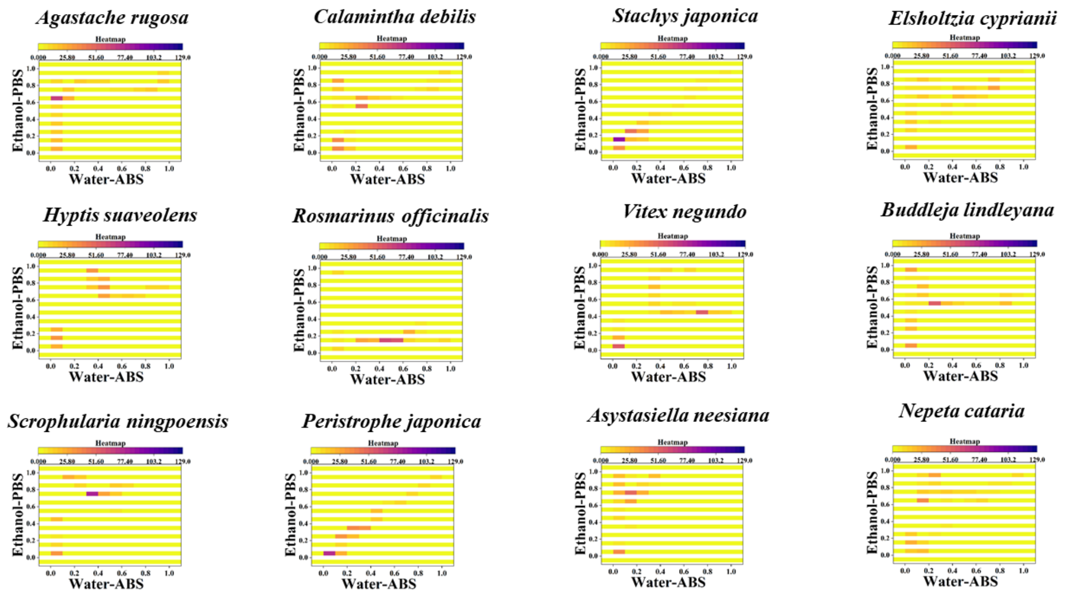

Figure S10. Heatmap of *Agastache rugosa, Calamintha debilis, Stachys japonica, Elsholtzia cyprianii, Hyptis suaveolens, Rosmarinus officinalis, Vitex negundo, Buddleja lindleyana, Scrophularia ningpoensis, Peristrophe japonica, Asystasiella neesiana, Nepeta cataria* combining the signals collected under PBS for the water extracts and under ABS for the ethanol extracts.
